# Mortality and losses to follow‐up among adolescents living with HIV in the IeDEA global cohort collaboration

**DOI:** 10.1002/jia2.25215

**Published:** 2018-12-13

**Authors:** Azar Kariminia, Matthew Law, Mary‐Ann Davies, Michael Vinikoor, Kara Wools‐Kaloustian, Valeriane Leroy, Andrew Edmonds, Catherine McGowan, Rachel Vreeman, Lee Fairlie, Samuel Ayaya, Marcel Yotebieng, Elom Takassi, Jorge Pinto, Adebola Adedimeji, Karen Malateste, Daisy M Machado, Martina Penazzato, Rohan Hazra, Annette H Sohn, on behalf of IeDEA

**Affiliations:** ^1^ The Kirby Institute UNSW Sydney Sydney Australia; ^2^ Centre for Infectious Disease Epidemiology and Research University of Cape Town Cape Town South Africa; ^3^ University of Alabama at Birmingham Birmingham AL USA; ^4^ Indiana University Indianapolis IN USA; ^5^ Inserm, U1027 Université Paul Sabatier Toulouse France; ^6^ The University of North Carolina at Chapel Hill Chapel Hill NC USA; ^7^ Vanderbilt University School of Medicine Nashville TN USA; ^8^ Wits Reproductive Health and HIV Institute Johannesburg South Africa; ^9^ Moi University College of Health Sciences Eldoret Kenya; ^10^ The Ohio State University Columbus OH USA; ^11^ CHU Sylvanus Olympio Lome Togo; ^12^ Federal University of Minas Gerais Belo Horizone Brazil; ^13^ Albert Einstein College of Medicine Bronx NY USA; ^14^ Inserm U1219 University of Bordeaux Bordeaux France; ^15^ Federal University of São Paulo São Paulo Brazil; ^16^ World Health Organization Geneva Switzerland; ^17^ Eunice Kennedy Shriver National Institute of Child Health and Human Development Bethesda MD USA; ^18^ TREAT Asia/amfAR The Foundation for AIDS Research Bangkok Thailand

**Keywords:** adolescents, mortality, lost to follow‐up, retention, global, HIV

## Abstract

**Introduction:**

We assessed mortality and losses to follow‐up (LTFU) during adolescence in routine care settings in the International epidemiology Databases to Evaluate AIDS (IeDEA) consortium.

**Methods:**

Cohorts in the Asia‐Pacific, the Caribbean, Central, and South America, and sub‐Saharan Africa (Central, East, Southern, West) contributed data, and included adolescents living with HIV (ALHIV) enrolled from January 2003 and aged 10 to 19 years (period of adolescence) while under care up to database closure (June 2016). Follow‐up started at age 10 years or the first clinic visit, whichever was later. Entering care at <15 years was a proxy for perinatal infection, while entering care ≥15 years represented infection acquired during adolescence. Competing risk regression was used to assess associations with death and LTFU among those ever receiving triple‐drug antiretroviral therapy (triple‐ART).

**Results:**

Of the 61,242 ALHIV from 270 clinics in 34 countries included in the analysis, 69% (n = 42,138) entered care <15 years of age (53% female), and 31% (n = 19,104) entered care ≥15 years (81% female). During adolescence, 3.9% died, 30% were LTFU and 8.1% were transferred. For those with infection acquired perinatally versus during adolescence, the four‐year cumulative incidences of mortality were 3.9% versus 5.4% and of LTFU were 26% versus 69% respectively (both *p *<* *0.001). Overall, there were higher hazards of death for females (adjusted sub‐hazard ratio (asHR) 1.19, 95% confidence interval (CI) 1.07 to 1.33), and those starting treatment at ≥5 years of age (highest asHR for age ≥15: 8.72, 95% CI 5.85 to 13.02), and in care in mostly urban (asHR 1.40, 95% CI 1.13 to 1.75) and mostly rural settings (asHR 1.39, 95% CI 1.03 to 1.87) compared to urban settings. Overall, higher hazards of LTFU were observed among females (asHR 1.12, 95% CI 1.07 to 1.17), and those starting treatment at age ≥5 years (highest asHR for age ≥15: 11.11, 95% CI 9.86 to 12.53), in care at district hospitals (asHR 1.27, 95% CI 1.18 to 1.37) or in rural settings (asHR 1.21, 95% CI 1.13 to 1.29), and starting triple‐ART after 2006 (highest asHR for 2011 to 2016 1.84, 95% CI 1.71 to 1.99).

**Conclusions:**

Both mortality and LTFU were worse among those entering care at ≥15 years. ALHIV should be evaluated apart from younger children and adults to identify population‐specific reasons for death and LTFU.

## 1 Introduction

UNAIDS estimates that there were 1.0 million female and 770,000 male adolescents living with HIV in 2017 (aidsinfo.unaids.org). The adolescent age group (10 to 19 years) represents a combination of young people who were perinatally infected with HIV and those more recently infected, often through high‐risk behaviours 1. Many adolescents are not accessing HIV treatment or have challenges with adherence and retention in care, with subsequent poor health outcomes 2, 3, 4, 5.

Although UNAIDS Global AIDS Monitoring protocols recommend the collection and reporting of data in detailed age groups, only 84 of 178 (47%) countries reported age‐disaggregated paediatric data (separating 10‐ to 14‐ and 15‐ to 19‐year olds) in 2016 6. The quality of the strategic information used to monitor the adolescent HIV epidemic and the impact of youth‐focused programmatic interventions could be enhanced by including routinely collected observational data from clinical and programme settings that are sufficiently detailed to be analysed by multiple categories (e.g. sex, age) and used to assess predictors of antiretroviral treatment (ART) outcomes. The objective of this analysis was to describe mortality and retention among a mixed population of adolescents living with HIV acquired perinatally as well as later in adolescence in routine care settings in low‐ and middle‐income countries in the International epidemiology Databases to Evaluate AIDS (IeDEA) cohort consortium.

## 2 Methods

### 2.1 Study population

IeDEA is a collaboration of clinical centres and research partners across seven global regions which was established in 2006 and is supported through the US National Institutes of Health (https://www.iedea.org/). For this analysis, data from 270 sites were included from six IeDEA regions (Asia‐Pacific, Central, East, West, and Southern Africa, the Caribbean and Central and South America (CCASAnet)), with data from Southern Africa separated into South Africa and the rest of Southern Africa due to variations in national paediatric HIV treatment guidelines (e.g. regarding use of protease inhibitors (PIs)) that resulted in differing treatment histories from other countries in that region. The analysis included all patients enrolled in HIV care at participating IeDEA sites from January 2003, and who had at least six months of potential follow‐up during adolescence (i.e. 10 to 19 years of age). Patients could have initiated antiretrovirals or remained antiretroviral‐naïve. The analysis database included data up to June 2016.

### 2.2 Ethics review

Each region secured local regulatory approvals for participation in this analysis, including reviews by local research and ethics regulatory bodies and, where required, national‐level approvals. Consent and assent requirements and procedures were regulated by the local regulatory bodies, and adherence to those standards was the responsibility of each site while being monitored and managed by regional coordinating centres. (https://www.iedea.org/regions/) 7, 8, 9.

### 2.3 Definitions and measurements

Adolescents were defined as those 10 up to 19 years of age and the analysis focused on this period of life. The beginning of follow‐up, referred to as the “baseline” time point, was the date of the 10th birthday for those who entered care before age 10, and the date of the first clinic visit for those who entered care at or after age 10. Follow‐up time ended and data were censored at whichever of the following came first: (1) death, (2) transfer out, (3) loss to follow‐up (LTFU), (4) turning 19 years of age, or (5) the closing date of the individual regional cohort database. The main outcomes of interest were death, LTFU and transfer that occurred in the year following the last clinic visit during the period of adolescence. Specifically, adolescents without evidence of contact with the clinic for more than 12 months were classified as LTFU with their follow‐up period ending 12 months after their last clinic contact. In addition, if a patient previously considered LTFU was subsequently known to have died or have been transferred (e.g. through updated reporting by their clinic), their outcomes were revised up to 24 months after their last clinic contact (and not beyond turning age 19 or database closure).

HIV disease stage was categorized as asymptomatic (CDC N or WHO 1), mild (CDC A or WHO 2), moderate (CDC B or WHO 3) and severe (CDC C or WHO 4). Weight and height measurements were converted to age‐ and sex‐adjusted z‐scores. For weight‐for‐age z‐scores, US National Center for Health Statistics and WHO International Growth Reference standards were used to allow for scoring children >10 years of age 10, 11. For height‐for‐age z‐score we used the WHO 2006/2007 Child Growth Standards 12, 13. Severe immunodeficiency was defined according to 2006 WHO global guidelines (e.g. <15% or <200 cells/mm^3^ for children ≥5 years old) 14.

For laboratory and clinical measurements, we used the closest values reported during a window of plus or minus three months from the baseline visit (i.e. at age 10 or the date of the first visit if entering care after age 10), with the pre‐baseline measurement used in the case of multiple values. At antiretroviral initiation, we used a testing window of three months before and one week after start (e.g. for CD4, viral load).

### 2.4 Statistical analysis

The analysis was restricted to assess outcomes during the period of adolescence. Adolescents entering care before age 15 years were compared to those with a first visit at or after age 15. Entry into care before age 15 was considered a proxy for those likely to have been infected perinatally or very early in life compared to those infected in older adolescence, predominantly assumed to be through risk behaviours and called the “late‐infected” 15, 16, 17. The term “late‐infected” was chosen to characterize the timing of HIV infection relative to the stage of adolescence (i.e. between 15 and 19 years of age). The selection of the age threshold is consistent with UNAIDS Global AIDS Monitoring methods where those 10 to 14 years of age are considered to be in early adolescence and those 15 to 19 years in late adolescence. In addition, infections acquired through risk behaviours are not modelled in the UNAIDS Spectrum model to occur among those entering HIV care before the age of 15 years 6. We conducted a sensitivity analysis to examine the impact of differentiating patients by age <10 versus ≥10 years at entry into care as a comparison 18.

To compare proportions, we used chi‐square tests, and we compared medians with the Mann–Whitney test. We used a cumulative incidence function to estimate the probabilities of death and LTFU during adolescence. In a subset of adolescents who had received ≥3 antiretrovirals as their initial treatment regimen, we conducted separate competing risks regression analyses based on Fine and Gray's proportional sub‐hazards model 19 to identify correlates of death (LTFU as a competing event) and correlates of LTFU (death as a competing event) from the start of triple‐drug ART. The following variables were included in the univariate analysis: age and calendar year at first triple‐drug ART, sex, facility level and facility setting (as defined by the site). CD4 count and weight‐for‐age z‐scores were included as time‐updated variables, and their values were carried forward if no subsequent measurements were recorded. In regression analyses, missing data were modelled as a separate category within each variable. We did not use multiple imputation to model missing data due to the relatively small numbers of covariates available, and the resulting lack of precision in imputation. To assess the robustness of our analyses to missing data, we undertook a sensitivity analysis based on subsets of patients with complete data. Variables were included in the multivariate model if they had a *p* < 0.2 in univariate analysis. We selected the final model using a backward elimination procedure and retained all variables in the model that had a *p* < 0.05. The adjusted subdistribution hazard ratios (asHR) were reported with their 95% confidence intervals (95% CI).

Management of the multiregional aggregated data and statistical analyses were performed at the Kirby Institute, UNSW Australia, using SAS version 9.4 (SAS Institute Inc., Cary, NC, USA) and Stata (StataCorp, STATA 14.0 for Windows, College Station, TX, USA).

## 3 Results

**Figure 1:**
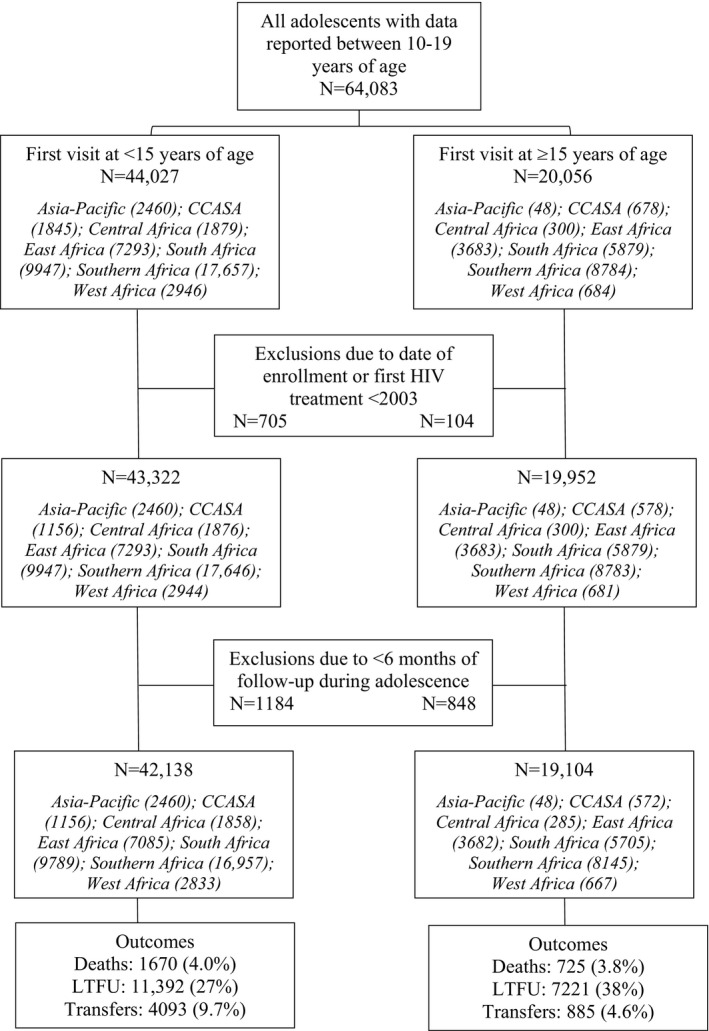
Flow diagram for analysis cohort by age at entry into HIV care (N = 61,242). CCASA, Caribbean, Central America, South America; LTFU, lost to follow‐up.

A total of 61,242 adolescents (61% female) were included from 270 sites in 34 countries (Figure 1): Asia‐Pacific (n = 2508, 4.1%; 16 sites), Central Africa (n = 2143, 3.5%; 15 sites), CCASAnet (n = 1728, 2.8%; 12 sites), East Africa (n = 10,767, 18%; 47 sites), South Africa (n = 15,494, 25%; 12 sites), Southern Africa (n = 25,102, 41%; 148 sites) and West Africa (n = 3500, 5.7%; 20 sites). Overall, 69% (n = 42,138) entered care before 15 years of age (perinatally infected) at a median age at first visit of 9.8 (interquartile range (IQR) 6.8 to 12.0) years (Table 1) and median duration of follow‐up during adolescence of 2.9 (IQR 1.4 to 5.0) years. Those entering care at or after age 15 (late‐infected) represented 31% (n = 19,104) of the adolescents, and had a median age at first visit of 17.5 (IQR 16.4 to 18.3) years and median duration of follow‐up during adolescence of 1.0 (IQR 0.9 to 1.6) year. Approximately one‐third of adolescents in CCASAnet (33%), and East (34%), South (37%), and Southern Africa (32%) were late‐infected, compared to 1.9% in the Asia‐Pacific, 13% in Central Africa and 19% in West Africa.

**Table 1:** Characteristics at analysis baseline^a^ for 61,242 patients who had a care visit at 10 to 19 years of age at an IeDEA site between 2003 and 2016

Characteristics^b^	At age 10 or first visit if first visit < age 15 N = 42,138 (68.8%)	At first visit if first visit ≥ age 15 N = 19,104 (31.2%)	*p*‐value
Sex			
Male	19,816 (47.0)	3638 (19.0)	<0.001
Female	22,137 (52.5)	15,420 (80.7)	
Unknown	185 (0.4)	46 (0.3)	
Age at first clinic visit (years)			
Median (IQR)	9.8 (6.8, 12.0)	17.5 (16.4, 18.3)	N/A
Mean (SD)	9.3 (3.48)	17.3 (1.14)	
CD4 count (cells/mm^3^)			
<200	6447 (15.3)	3764 (19.7)	<0.001
200 to 349	4087 (9.7)	3360 (17.6)	
350 to 499	3668 (8.7)	2508 (13.1)	
≥500	11,176 (26.5)	3617 (18.9)	
Unknown	16,760 (39.8)	5855 (30.7)	
Median (IQR)	435 (196, 745)	329 (178, 521)	<0.001
Mean (SD)	513 (405.1)	377 (274.3)	<0.001
HIV viral load, copies/mL			
<50	3026 (7.2)	227 (1.2)	<0.001
50 to 399	1535 (3.6)	132 (0.7)	
400 to 999	472 (1.1)	42 (0.2)	
1000 to 9999	837 (2.0)	157 (0.8)	
≥10,000	2199 (5.2)	483 (2.5)	
Unknown	34,069 (80.9)	18,063 (94.6)	
Median log_10_ (IQR) HIV‐RNA	2.4 (1.6, 4.2)	3.9 (1.9, 4.9)	<0.001
Mean (SD)	2.8 (1.60)	3.6 (1.58)	<0.001
WHO/CDC clinical stage			
WHO stage 1/CDC stage N	1759 (4.2)	1808 (9.5)	<0.001
WHO stage 2/CDC stage A	2613 (6.2)	885 (4.6)	
WHO stage 3/CDC stage B	2638 (6.3)	798 (4.2)	
WHO stage 4/CDC stage C	1872 (4.4)	267 (1.4)	
Not documented	33,256 (78.9)	15,346 (80.3)	
Weight‐for‐age z‐score			
<−3	7179 (17.0)	2079 (10.9)	<0.001
−3 ≤ to <−2	6644 (15.8)	1401 (7.3)	
−2 ≤ to <−1	9051 (21.5)	2353 (12.3)	
≥−1	7664 (18.2)	6690 (35.0)	
Unknown	11,600 (27.5)	6581 (34.5)	
Median (IQR)	−1.9 (−2.9, −1.0)	−0.9 (−2.2, −0.1)	<0.001
Mean (SD)	−2.1 (1.63)	−1.3 (1.99)	<0.001
Height‐for‐age z‐score			
<−3	5254 (12.5)	851 (4.5)	<0.001
−3 ≤ to <−2	6896 (16.4)	1348 (7.1)	
−2 ≤ to <−1	7642 (18.1)	3138 (16.4)	
≥−1	6422 (15.2)	5136 (26.9)	
Unknown	15,924 (37.8)	8631 (45.2)	
Median (IQR)	−1.9 (−2.8, −1.0)	−1.0 (−1.9, −0.3)	<0.001
Mean (SD)	−1.9 (1.42)	−1.1 (1.35)	<0.001
Timing of antiretrovirals^c^			
Started before baseline	17,420 (41.3)	164 (0.9)	<0.001
Started at baseline	4982 (11.8)	3232 (16.9)	
Type of regimen^c^^,^ d			
3‐ART‐NNRTI	18,389 (82.1)	2822 (83.1)	<0.001
3‐ART‐PI	1489 (6.7)	147 (4.3)	
3‐ART‐NNRTI/PI based	55 (0.3)	2 (0.1)	
3‐ART‐other	52 (0.2)	11 (0.3)	
Mono/dual	2417 (10.8)	414 (12.2)	
Most frequent antiretroviral regimen^c^			
3TC/d4T/EFV	4105 (18.3)	191 (5.6)	<0.001
3TC/d4T/NVP	3759 (16.8)	265 (7.8)	
3TC/AZT/NVP	3362 (15.0)	184 (5.4)	
3TC/AZT/EFV	2418 (10.8)	156 (4.6)	
ABC/3TC/EFV	2264 (10.1)	53 (1.6)	
ABC/3TC/NVP	1344 (6.0)	16 (0.5)	
FTC/EFV/TDF	313 (1.4)	1057 (31.1)	
3TC/EFV/TDF	314 (1.4)	653 (19.2)	
3TC/NVP/TDF	47 (0.2)	153 (4.6)	
Others^e^	4476 (20.0)	668 (19.7)	
Duration on antiretrovirals, years^f^			
<1	3765 (21.6)	91 (55.5)	<0.001
1 to 2	5764 (33.1)	27 (16.5)	
≥3	7891 (45.3)	46 (28.1)	
Median (IQR)	2.7 (1.2, 4.6)	0.6 (0.1, 3.9)	<0.001
Mean (SD)	3.1 (2.29)	2.1 (2.55)	<0.001

Data are presented as n (%) unless otherwise noted. We used the 1977 WHO growth curve for weight‐for‐age z‐score (more recent weight curves are limited to children age ≤10 years) and the 2006/2007 WHO growth curve for height‐for‐age z‐score. 3‐ART, antiretroviral therapy regimen of three or more antiretrovirals; IQR, interquartile range; NNRTI, non‐nucleoside reverse transcriptase inhibitor; PI, protease inhibitor; mono/dual, single or two drugs; SD, standard deviation.

^a^Baseline was the date of the 10th birthday for those who entered care before age 10, and the date of the first clinic visit for those who entered care at or after age 10. ^b^IeDEA regions utilize a common data exchange standard for harmonizing data for use in multiregional analyses that includes formats and categorizations for specific data variables that are available at iedeades.org. ^c^This only includes those who were on antiretrovirals at baseline. Those who started before baseline and stopped before baseline and those who started antiretrovirals after baseline were not included. ^d^3‐ART represents triple‐drug regimens. The drug class following that term denotes where one of the drugs included either an NNRTI, PI, or both classes; “other” represents triple‐drug regimens without an NNRTI or PI. Non‐3‐ART represents regimens with fewer than three individual antiretroviral drugs. ^e^Includes other triple‐drug and mono/dual antiretroviral combinations. ^f^Duration has been calculated for adolescents who started antiretrovirals before baseline and were still on them at baseline.

At baseline (age 10 years or first clinic visit if entering care later), antiretrovirals had already been started by 41% of perinatally infected adolescents and 0.9% of late‐infected adolescents. The median CD4 count was 435 (IQR 196 to 745) cells/mm^3^ for those perinatally infected and 329 (IQR 178 to 521) cells/mm^3^ for the late‐infected; higher proportions of perinatally infected were severely underweight (17% vs. 11%, *p *<* *0.001) and severely stunted (13% vs. 4.5%, *p *<* *0.001) with z‐scores <−3 compared to late‐infected adolescents. At baseline, HIV viral load was infrequently available in both groups (19% vs. 5.4%). Of those with a viral load measurement at baseline, 38% of the perinatally infected and 22% of the late‐infected were undetectable.

### 3.1 Antiretroviral use

A total of 44,922 adolescents ever initiated any combination of antiretroviral drugs by the end of the follow‐up period (84% perinatally vs. 51% late‐infected; Table S1). Median CD4 percent at antiretroviral initiation was 12% (IQR 6% to 18%) among the perinatally infected and 14% (IQR 7% to 23%) among late‐infected adolescents (*p *<* *0.001). Perinatally infected adolescents had a greater degree of severe immunodeficiency (59% vs. 51%; *p *<* *0.001) and were more likely to be severely underweight (22% vs. 13%; *p *<* *0.001) and stunted (16% vs. 4.6%; *p *<* *0.001) at antiretroviral start. The median lifetime duration of antiretroviral use was 4.8 (IQR 2.4 to 7.3) years among those entering care before age 15 years, and 1.1 (IQR 0.8 to 1.8) years for those entering at ≥15 years.

### 3.2 Patient outcomes

**Figure 2:**
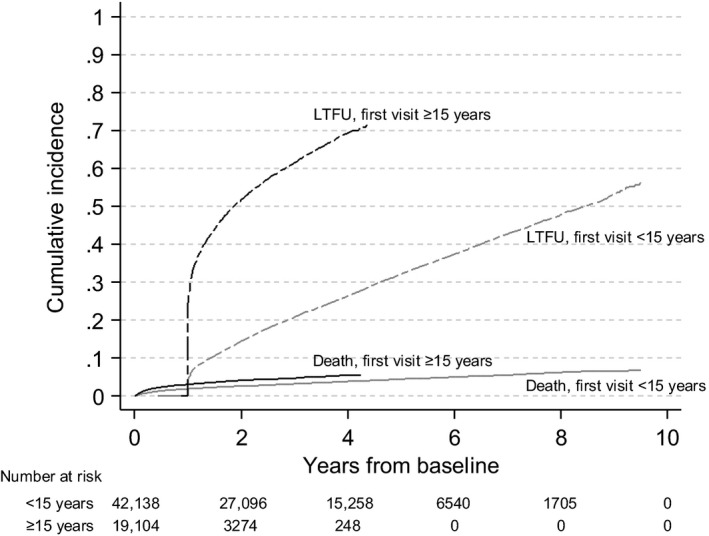
Estimated cumulative incidences of death and loss to follow‐up using competing risk methods, among adolescents (10 to 19 years) enrolled at 270 IeDEA clinical sites from 2003 to 2016, by age at first clinic visit (n = 61,242).

During the adolescent follow‐up period, 3.9% died, 30% were LTFU and 8.1% were transferred. Separated by age at cohort entry, among those entering care before age 15 years, 4.0% died, 27% were LTFU, and 9.7% transferred (Table S2). For those entering care at or after age 15 years, 3.8% died, 38% were LTFU and 4.6% were transferred. These data include 62 adolescents living with HIV (ALHIV) who were recategorized from LTFU to dead and 98 recategorized to transferred between 12 and 24 months after most recent clinic contact (and before age 19 or database closure). Among perinatally infected adolescents, the four‐year cumulative incidence of death was 3.9% and of LTFU was 26%, while for late‐infected adolescents it was 5.4% for death and 69% for LTFU; both outcomes were significantly higher in late‐infected adolescents (*p *<* *0.001) (Figure 2).

In the sensitivity analysis where age at entry into care was redefined as <10 years versus ≥10 years, the median CD4 at baseline for the group entering care at <10 years of age (n = 22,168) was 673 (IQR 429 to 949) cells/mm^3^. The proportion of adolescents entering care before age 10 years who died was 1.9%, compared to 4.0% using the age 15 threshold, and the proportion who were LTFU was 18% compared to 27% (Table S3). In addition, of the 19,970 adolescents entering care between age 10 and 14 years, 1247 (6.2%) died and 7469 (37%) were LTFU by the age of 19 years.

The multivariate regression model restricted to those who received triple‐drug ART as their initial antiretroviral regimen showed that there was an increase in the hazard rate of death for those starting treatment at older ages compared to those <5 years of age (5 to 9 years adjusted subdistribution hazard ratios asHR 2.59, 95% CI 1.74 to 3.85; 10 to 14 years asHR 6.93, 95% CI 4.69 to 10.22; ≥15 years asHR 8.72, 95% CI 5.85 to 13.02) (Table 2). The hazard was higher for females (asHR 1.19, 95% CI 1.07 to 1.33), and those receiving care in mostly urban (asHR 1.40, 95% CI 1.13 to 1.75) and mostly rural settings (asHR 1.39, 95% CI 1.03 to 1.87) compared to urban settings. The hazard of death was lower for those with higher CD4 count, better weight‐for‐age z‐scores, receiving care at a district hospital and in rural settings compared to health centres and in urban settings, with a later year of starting ART, and for cohorts from the Asia‐Pacific, Central Africa, East Africa, and South Africa compared to Southern Africa. Hazard rates of death were lowest overall among adolescents with a current CD4 ≥500 cells/mm^3^ compared to <200 cells/mm^3^ (asHR 0.12, 95% CI 0.10 to 0.15), a weight‐for‐age z‐score ≥−2 compared to <−3 (asHR 0.22, 95% CI 0.19 to 0.25), initiating ART between 2011 and 2016 compared to 2003 and 2006 (asHR 0.36, 95% CI 0.31 to 0.43), or receiving care in South Africa compared to Southern Africa (asHR 0.45, 95% CI 0.36 to 0.57).

**Table 2:** Factors associated with death during the adolescent period (10 to 19 years of age) in 39,262 patients who received ≥3 antiretroviral drugs as initial HIV treatment regimens

Characteristics	Total (N = 39,262)	Deaths (N = 1518)	Univariate		Multivariate	
			asHR (95% CI)	*p*‐value	asHR (95% CI)	*p*‐value
Sex						
Male	16,475	681	1.00		1.00	
Female	22,640	832	0.99 (0.90, 1.10)	0.899	1.19 (1.07, 1.33)	0.001
Age at ≥3‐drug ART (years)						
<5	3006	26	1.00		1.00	
5 to 9	11,923	248	3.05 (2.05, 4.53)	<0.001	2.59 (1.74, 3.85)	<0.001
10 to 14	14,522	844	11.28 (7.73, 16.45)	<0.001	6.93 (4.69, 10.22)	<0.001
≥15	9811	400	11.26 (7.67, 16.52)	<0.001	8.72 (5.85, 13.02)	<0.001
Current CD4 count (cells/mm^3^)^a^						
<200	‐	913	1.00			
200 to 349	‐	162	0.20 (0.17, 0.23)	<0.001	0.27 (0.23, 0.32)	<0.001
350 to 499	‐	121	0.15 (0.13, 0.18)	<0.001	0.23 (0.19, 0.28)	<0.001
≥500	‐	170	0.06 (0.05, 0.07)	<0.001	0.12 (0.10, 0.15)	<0.001
Current weight‐for‐age z score^a^						
<−3	‐	834	1.00		1.00	
−3 ≤ to <−2	‐	199	0.22 (0.19, 0.26)	<0.001	0.34 (0.29, 0.40)	<0.001
≥−2	‐	325	0.12 (0.11, 0.14)	<0.001	0.22 (0.19, 0.25)	<0.001
Facility level						
Health centre	16,068	532	1.00			
District hospital	6708	232	1.11 (0.95, 1.30)	0.174	0.76 (0.64, 0.91)	0.003
Regional, provincial, or university hospital	13,214	635	1.31 (1.17,1.47)	<0.001	1.02 (0.86, 1.22)	0.800
Facility setting						
Urban	16,141	750	1.00		1.00	
Mostly urban	4857	241	1.10 (0.95, 1.30)	0.184	1.40 (1.13, 1.75)	0.002
Mostly rural	2758	114	1.04 (0.85, 1.26)	0.723	1.39 (1.03, 1.87)	0.032
Rural	12,184	291	0.57 (0.49, 0.65)	<0.001	0.76 (0.63, 0.91)	0.003
Region						
Southern Africa	11,640	528	1.00		1.00	
Asia‐Pacific	2295	85	0.62 (0.49, 0.78)	0.001	0.54 (0.39, 0.75)	0.001
Caribbean, Central and South America	1473	114	1.40 (1.15, 1.72)	0.001	0.96 (0.72, 1.26)	0.748
Central Africa	1660	54	0.59 (0.45, 0.78)	<0.001	0.68 (0.48, 0.95)	0.026
East Africa	7476	303	0.90 (0.78, 1.04)	0.160	0.49 (0.37, 0.65)	<0.001
South Africa	11,574	202	0.35 (0.29, 0.41)	<0.001	0.45 (0.36, 0.57)	<0.001
West Africa	3144	232	1.37 (1.18, 1.60)	<0.001	1.02 (0.80, 1.30)	0.859
Year of first ≥3‐drug ART						
2003 to 2006	8560	554	1.00		1.00	
2007 to 2010	14,553	659	0.83 (0.74, 0.92)	0.001	0.69 (0.61, 0.78)	<0.001
2011 to 2016	16,149	305	0.49 (0.43, 0.57)	<0.001	0.36 (0.31, 0.43)	<0.001

Loss to follow‐up (n = 9131) was a competing event for death in this analysis and death was a competing event for loss to follow‐up. Total numbers include missing values (not shown in the table). Missing values were included as a separate category in all analyses. 95% CI, 95% confidence interval; asHR, adjusted subdistribution hazard ratio.

a CD4 count and weight‐for‐age z score were considered time‐dependent variables. Total number was not given as adolescents moved between categories.

Increased hazard rates of LTFU among adolescents who received triple‐drug ART as their initial antiretroviral regimen were associated with female sex (asHR 1.12, 95% CI 1.07 to 1.17), older age at ART start compared to <5 years (5 to 9 years asHR 2.59, 95% CI 2.32 to 2.88; 10 to 14 years asHR 6.11, 95% CI 5.49 to 6.81; ≥15 years asHR 11.11, 95% CI 9.86 to 12.53), receiving care at a district hospital compared to a health centre (asHR 1.27, 95% CI 1.18, 1.37), receiving care in rural compared to urban settings (asHR 1.21, 95% CI 1.13, 1.29), receiving care in East Africa (asHR 1.14, 95% CI 1.01 to 1.28), South Africa (asHR 1.75, 95% CI 1.63 to 1.88), or CCASAnet (asHR 2.99, 95% CI 2.65 to 3.36) compared to Southern Africa, and starting triple‐drug ART after 2006 (highest asHR for 2011 to 2016 1.84, 95% CI 1.71 to 1.99) (Table 3). In contrast, lower hazard rates of LTFU were associated with CD4 count ≥350 cells/mm^3^ (lowest asHR for ≥500 0.65, 95% CI 0.61 to 0.69), receiving care in regional, provincial, or university hospitals compared to health centres (asHR 0.63, 95% CI 0.58 to 0.68), receiving care in mostly urban and mostly rural compared to urban settings (lowest asHR for mostly urban 0.71, 95% CI 0.62 to 0.81), and receiving care in the Asia‐Pacific compared to Southern Africa (asHR 0.19, 95% CI 0.14 to 0.25).

**Table 3:** Factors associated with LTFU during the adolescent period (10 to 19 years of age) in 39,262 patients who received ≥3 antiretroviral drugs as initial HIV treatment regimens

Characteristics	Total (N = 39,262)	LTFU (N = 9131)	Univariate		Multivariate	
			asHR (95% CI)	*p*‐value	asHR (95% CI)	*p*‐value
Sex						
Male	16,475	3753	1.00		1.00	<0.001
Female	22,640	5374	1.33 (1.27, 1.38)	<0.001	1.12 (1.07, 1.17)	
Age at ≥3‐drug ART (years)						
<5	3006	304	1.00		1.00	
5 to 9	11,923	2198	3.17 (2.84, 3.54)	<0.001	2.59 (2.32, 2.88)	<0.001
10 to 14	14,522	4142	9.90 (8.91, 11.00)	<0.001	6.11 (5.49, 6.81)	<0.001
≥15	9811	2487	24.54 (21.94, 27.46)	<0.001	11.11 (9.86, 12.53)	<0.001
Current CD4 count (cells/mm^3^)^a^						
<200	‐	1824	1.00		1.00	
200 to 349	‐	1489	0.92 (0.86, 0.99)	0.017	1.01 (0.94, 1.08)	0.777
350 to 499	‐	1143	0.57 (0.53, 0.61)	<0.001	0.72 (0.67, 0.78)	<0.001
≥500	‐	3076	0.35 (0.33, 0.37)	<0.001	0.65 (0.61, 0.69)	<0.001
Current weight‐for‐age z score^a^						
<−3	‐	1448	1.00			
−3 ≤ to <−2	‐	1046	0.64 (0.59, 0.69)	<0.001	0.93 (0.86, 1.01)	0.079
≥−2	‐	3252	0.65 (0.61, 0.69)	<0.001	0.94 (0.88, 1.01)	0.087
Facility level						
Health centre	16,068	4747	1.00		1.00	
District hospital	6708	1527	0.84 (0.79, 0.89)	<0.001	1.27 (1.18, 1.37)	<0.001
Regional, provincial, or university hospital	13,214	2091	0.37 (0.35, 0.39)	<0.001	0.63 (0.58, 0.68)	<0.001
Facility location						
Urban	16,141	3185	1.00			
Mostly urban	4857	545	0.58 (0.53, 0.64)	<0.001	0.71 (0.62, 0.81)	<0.001
Mostly rural	2758	401	0.99 (0.90, 1.10)	0.914	0.75 (0.64, 0.87)	<0.001
Rural	12,184	4223	2.70 (2.58, 2.82)	<0.001	1.21 (1.13, 1.29)	<0.001
Region						
Southern Africa	11,640	2776	1.00		1.00	
Asia‐Pacific	2295	49	0.04 (0.03, 0.06)	<0.001	0.19 (0.14, 0.25)	<0.001
Caribbean, Central and South America	1473	649	1.21 (1.12, 1.31)	0.822	2.99 (2.65, 3.36)	<0.001
Central Africa	1660	266	0.42 (0.37, 0.47)	<0.001	0.93 (0.80, 1.08)	0.347
East Africa	7476	1152	0.61 (0.57, 0.66)	<0.001	1.14 (1.01, 1.28)	0.028
South Africa	11,574	3850	1.22 (1.16, 1.28)	<0.001	1.75 (1.63, 1.88)	0.001
West Africa	3144	389	0.31 (0.28, 0.35)	<0.001	0.93 (0.81, 1.06)	0.288
Year of first ≥3‐drug ART						
2003 to 2006	8560	1940	1.00		1.00	
2007 to 2010	14,553	3689	2.11 (1.99, 2.24)	<0.001	1.24 (1.16, 1.32)	<0.001
2011 to 2016	16,149	3502	5.36 (5.03, 5.72)	<0.001	1.84 (1.71, 1.99)	<0.001

Death (n = 1518) was a competing event for LTFU in this analysis and loss to follow‐up was a competing event for death. Total numbers include missing values (not shown in the table). Missing values were included as a separate category in all analyses. 95% CI, 95% confidence interval; asHR, adjusted subdistribution hazard ratio; LTFU, loss to follow‐up.

a CD4 count and weight‐for‐age z score were considered time‐dependent variables. Total number was not given as adolescents moved between categories.

The sensitivity analyses excluding missing data gave qualitatively very similar results for most covariates (data not shown). The only exceptions were in mortality analyses, where survival was no longer improved in Central Africa (asHR in sensitivity analysis of 1.10 95% CI 0.71 to 1.70, compared with asHR in main analysis of 0.68 95% 0.48 to 0.95), or in rural settings (asHR in sensitivity analysis of 1.02 95% CI 0.82 to 1.27, compared with asHR in main analysis of 0.76 95% 0.63 to 0.91). Importantly, results for individual‐level covariates such as age, sex and CD4 count, were qualitatively the same.

## 4 Discussion

This is the first IeDEA multiregional cohort analysis to reflect the complexity of the mixed adolescent HIV epidemic including both individuals with perinatally acquired HIV and those infected later. Among the 61,242 adolescents in our analysis, 69% entered care before the age of 15 (our proxy for perinatally acquired infection), but not until a median age of 9.8 years. While the cumulative incidence of both mortality and LTFU were higher among those entering care at ≥15 years (our proxy for infection acquired later during adolescence), the qualitative differences in mortality over this period were small. However, the sensitivity analysis demonstrated a higher burden of mortality among those perinatally infected adolescents who did not enter care until 10 to 14 years of age. Our overall four‐year cumulative incidence of death of 4.2% compares to the post‐ART mortality incidence rate of 0.97 per 100 person‐years among children five to nine years of age in IeDEA 20, and rates among youth starting ART during the ages of 15 to 24 years from 0.8 per 100 in Nigeria up to 13.5 per 100 in Tanzania in a seven‐African country analysis 2.

From our regression model, most factors found to be protective against death among those who started treatment with triple‐drug ART were consistent with other studies (e.g. better immune control, higher weight‐for‐age z‐score) 4, 21, 22. Any CD4 category ≥200 cells/mm^3^ or weight‐for‐age z‐score better than or equal to −3 was highly protective; as was starting triple‐drug ART after 2011, which may reflect scale‐up and quality improvement of paediatric HIV programmes and broadening treatment access in our settings 23, 24. Among those who had not received treatment, the median duration of follow‐up during adolescence was only one year and 52% were LTFU, making reliable ascertainment of mortality difficult in this sub‐group. The associations with regional cohort may reflect variations in national infrastructures for HIV and availability of other supportive healthcare services in the context of background country development 2, 25, 26, 27.

The overall high cumulative incidence of LTFU was concerning. Losses among those presenting to care during late adolescence rose sharply starting in the first year of follow‐up, whereas losses among the perinatally infected steadily increased over time. The four‐year cumulative incidence of LTFU during adolescence was 26% for the perinatally infected and almost three times that at 69% for the late‐infected. This compares to data from adolescents and young adults 15 to 24 years of age at treatment initiation in seven African countries, where LTFU ranged from 7.1% in Uganda to 30% in Tanzania 2. The rapid early losses are also consistent with levels of LTFU, approximately 30%, documented in prevention of mother‐to‐child HIV transmission Option B+ programmes 28, 29. While we did not have access to pregnancy data to confirm whether young women entered HIV care through antenatal care, being female was a risk factor for LTFU. While current universal treatment recommendations may reduce the early LTFU that was previously associated with delays related to CD4 testing 30, 31, 32, there are also studies among adolescents and adults reporting greater attrition among those started on ART at higher CD4 counts who have not experienced clinical disease progression 33, 34, 35.

The associations between LTFU and older age, as well as later year at cohort entry, may be related to having less time to return to care after an interruption (i.e. patient churn), survival bias among those who started treatment as younger children, or the poorer retention often seen among older adolescents and young adults 2, 36, 37, 38, 39. Receiving care in rural settings was associated with higher LTFU, but protective against death, which may suggest under ascertainment of mortality in rural areas where out migration in sub‐Saharan Africa has been common 40, 41. The reasons for the associations with regional cohort are unclear, and could be due to varying proportions of perinatally infected youth, local patient case mix, or other socio‐economic or demographic factors 18, 28, 42, 43. In addition, regional cohorts with sites within areas with a high density of ART programmes may see more “silent transfers,” where patients move between clinics without formal referrals.

The median baseline CD4 count for the group entering care between 15 and 19 years was unexpectedly low at 329 cells/mm^3^, implying that there may have been perinatally infected adolescents mixed into this group who entered care at older ages with advanced HIV disease. This may reflect the health status of older slow progressors among perinatal adolescent survivors or the potential contribution of rapid disease progressors among those infected during adolescence. Age alone may be insufficient to avoid misclassification, and more complex algorithms to assess combinations of routinely available variables (e.g. weight, height and CD4 count) would help to disaggregate data 15.

Our analysis was limited by the use of routinely collected clinical data, which were incomplete. We included children with missing data in analyses using missing value categories in covariates, which has the advantage of maximising the sample size. Sensitivity analyses which excluded missing data gave very similar results, but did result in some changes in differences in survival between regions and facility setting. These site‐level covariates should be interpreted especially cautiously. Beyond variations in clinical resources and programme policies, some of the regions had more older female adolescents enrolled, which may have been associated with antenatal care programmes where early LTFU rates have been high 28. Our focus on those 10 to 19 years of age results in a survivor bias, as individuals who were perinatally infected would have had to survive childhood in order to be eligible for inclusion. In addition, our age proxy for perinatally acquired infection may have miscategorized those presenting very late to care as being infected during adolescence. Restricting inclusion in the analysis to those with at least six months of data may have resulted in underreporting of LTFU. While we allowed for randomly collected tracing data to reclassify outcomes, we did not systematically adjust for the risk of unascertained mortality among those LTFU.

## 5 Conclusions

In this global analysis of adolescent outcomes in IeDEA, 3.9% of ALHIV were reported to have died and 30% were LTFU, with both rates higher in those entering care after age 15. However, those entering care between 10 and 15 years were at higher risk of death than those in care before age 10, reflecting the severe immunodeficiency associated with delayed diagnoses. Greater prioritization of adolescents for clinical and social support is urgently needed to retain youth in HIV treatment programmes as they transition through adolescence into adult life.

## Competing interest

AHS reports support to her institution for travel and grants from ViiV Healthcare, and ML reports unrestricted grants from Boehringer Ingelhiem, Gilead Sciences, Merck Sharp & Dohme, Bristol‐Myers Squibb, Janssen‐Cilag, ViiV HealthCare, consultancy fees from Gilead Sciences, and DSMB sitting fees from Sirtex Pty Ltd. Other authors have no conflicts to report.

## Authors’ contributions

AK, ML, and AHS developed and wrote the analysis concept and analysis plan. AK, ML, MAD, KWW, VL, AE, CM, RV, SA, MY, MP, RH, AHS revised and finalized the concept. AK, ML, MAD, MV, KWW, VL, AE, CM, RV, LF, SA, MY, ET, JP, AA, KM, DMM, and AHS were involved with the collection, preparation, and/or submission of the source data for the analysis. AK and ML conducted the analysis. AK and AHS wrote the paper. All co‐authors were involved with revisions of the paper, and read and approved the final version.

## Supporting information


**Table S1.** Characteristics at antiretroviral initiation for 44,922 adolescents who had a care visit at 10 to 19 years of age at an IeDEA site between 2003 and 2016
**Table S2**. Outcomes*, by region and age at first clinic visit (Group A, first visit <15 years of age; Group B, first visit ≥15 years of age)
**Table S3.** Outcomes*, by region and age at first clinic visit (Group A, first visit <10 years of age; Group B, first visit ≥10 years of age)

## Appendix 1 IeDEA global investigator acknowledgements by region

### IeDEA Asia‐Pacific

The TREAT Asia Pediatric HIV Observational Database is an initiative of TREAT Asia, a programme of amfAR, The Foundation for AIDS Research, with support from the US National Institutes of Health's National Institute of Allergy and Infectious Diseases, the Eunice Kennedy Shriver National Institute of Child Health and Human Development, National Cancer Institute, National Institute of Mental Health, and National Institute on Drug Abuse as part of the International Epidemiology Databases to Evaluate AIDS (IeDEA; U01AI069907). The Kirby Institute is funded by the Australian Government Department of Health and Ageing, and is affiliated with the Faculty of Medicine, UNSW Australia. The content of this publication is solely the responsibility of the authors and does not necessarily represent the official views of any of the governments or institutions mentioned above.

#### The TREAT Asia Pediatric HIV Network

PS Ly*, and V Khol, National Centre for HIV/AIDS, Dermatology and STDs, Phnom Penh, Cambodia; J Tucker, New Hope for Cambodian Children, Phnom Penh, Cambodia; N Kumarasamy*, and E Chandrasekaran, YRGCARE Medical Centre, CART CRS, Chennai, India; DK Wati*, D Vedaswari, and IB Ramajaya, Sanglah Hospital, Udayana University, Bali, Indonesia; N Kurniati*, and D Muktiarti, Cipto Mangunkusumo – Faculty of Medicine Universitas Indonesia, Jakarta, Indonesia; SM Fong*, M Lim, and F Daut, Hospital Likas, Kota Kinabalu, Malaysia; NK Nik Yusoff*&, and P Mohamad, Hospital Raja Perempuan Zainab II, Kelantan, Malaysia; TJ Mohamed* and MR Drawis, Pediatric Institute, Hospital Kuala Lumpur, Kuala Lumpur, Malaysia; R Nallusamy*, and KC Chan, Penang Hospital, Penang, Malaysia; T Sudjaritruk*, V Sirisanthana, and L Aurpibul, Department of Pediatrics, Faculty of Medicine, and Research Institute for Health Sciences, Chiang Mai University, Chiang Mai, Thailand; R Hansudewechakul*, P Ounchanum, S Denjanta, and A Kongphonoi, Chiangrai Prachanukroh Hospital, Chiang Rai, Thailand; P Lumbiganon*†, P Kosalaraksa, P Tharnprisan, and T Udomphanit, Division of Infectious Diseases, Department of Pediatrics, Faculty of Medicine, Khon Kaen University, Khon Kaen, Thailand; G Jourdain, PHPT‐IRD UMI 174 (Institut de recherche pour le développement and Chiang Mai University), Chiang Mai, Thailand; T Puthanakit*, S Anugulruengkit, W Jantarabenjakul and R Nadsasarn, Department of Pediatrics, Faculty of Medicine and Research Unit in Pediatric and Infectious Diseases, Chulalongkorn University, Bangkok, Thailand; K Chokephaibulkit*, K Lapphra, W Phongsamart, and S Sricharoenchai, Department of Pediatrics, Faculty of Medicine Siriraj Hospital, Mahidol University, Bangkok, Thailand; KH Truong*, QT Du, and CH Nguyen, Children's Hospital 1, Ho Chi Minh City, Vietnam; VC Do*, TM Ha, and VT An Children's Hospital 2, Ho Chi Minh City, Vietnam; LV Nguyen*, DTK Khu, AN Pham, and LT Nguyen, National Hospital of Pediatrics, Hanoi, Vietnam; ON Le, Worldwide Orphans Foundation, Ho Chi Minh City, Vietnam; AH Sohn*, JL Ross, and C Sethaputra, TREAT Asia/amfAR – The Foundation for AIDS Research, Bangkok, Thailand; MG Law* and A Kariminia, The Kirby Institute, UNSW Sydney, Sydney, Australia; (*Steering Committee members; †Current Steering Committee Chair; &co‐Chair).

#### Caribbean, Central, and South America (CCASAnet)

This work was supported by the NIH‐funded Caribbean, Central and South America network for HIV epidemiology (CCASAnet), a member cohort of the International Epidemiology Databases to Evaluate AIDS (leDEA) (U01AI069923). This award is funded by the following institutes: Eunice Kennedy Shriver National Institute Of Child Health & Human Development (NICHD), National Cancer Institute (NCI), National Institute Of Allergy And Infectious Diseases (NIAID), National Institute Of Mental Health (NIMH), and the Office Of The Director, National Institutes Of Health (OD).

**Fundación Huésped, Argentina:** Pedro Cahn, Carina Cesar, Valeria Fink, Omar Sued, Emanuel Dell'Isola, Hector Perez, Jose Valiente, Cleyton Yamamoto.

**Instituto Nacional de Infectologia‐Fiocruz, Brazil:** Beatriz Grinsztejn, Valdilea Veloso, Paula Luz, Raquel de Boni, Sandra Cardoso Wagner, Ruth Friedman, Ronaldo Moreira.

**Universidade Federal de Minas Gerais, Brazil:** Jorge Pinto, Flavia Ferreira, Marcelle Maia.

**Universidade Federal de São Paulo, Brazil:** Regina Célia de Menezes Succi, Daisy Maria Machado, Aida de Fátima Barbosa Gouvêa.

**Fundación Arriarán, Chile**: Marcelo Wolff, Claudia Cortes, Maria Fernanda Rodriguez, Gladys Allendes.

**Les Centres GHESKIO, Haiti:** Jean William Pape, Vanessa Rouzier, Adias Marcelin, Christian Perodin.

**Hospital Escuela Universitario, Honduras**: Marco Tulio Luque.

**Instituto Hondureño de Seguridad Social, Honduras:** Denis Padgett.

**Instituto Nacional de Ciencias Médicas y Nutrición Salvador Zubirán, Mexico**: Juan Sierra Madero, Brenda Crabtree Ramirez, Paco Belaunzaran, Yanink Caro Vega.

**Instituto de Medicina Tropical Alexander von Humboldt, Peru**: Eduardo Gotuzzo, Fernando Mejia, Gabriela Carriquiry.

**Vanderbilt University Medical Center, USA:** Catherine C McGowan, Bryan E Shepherd, Timothy Sterling, Karu Jayathilake, Anna K Person, Peter F Rebeiro, Mark Giganti, Jessica Castilho, Stephany N Duda, Fernanda Maruri, Hilary Vansell.

### Central Africa (CA‐IeDEA)

Research reported in this publication was supported by the National Institute of Allergy and Infectious Diseases of the National Institutes of Health under Award Number U01AI096299 (PI: Anastos and Nash). The content is solely the responsibility of the authors and does not necessarily represent the official views of the National Institutes of Health.

#### Site investigators and cohorts

Nimbona Pélagie, ANSS, Burundi; Patrick Gateretse, Jeanine Munezero, Valentin Nitereka, Théodore Niyongabo, Christelle Twizere, Centre National de Reference en Matiere de VIH/SIDA, Burundi; Hélène Bukuru, Thierry Nahimana, CHUK, Burundi; Jérémie Biziragusenyuka, Risase Scholastique Manyundo, HPRC, Burundi; Tabeyang Mbuh, Kinge Thompson Njie, Edmond Tchassem, Kien‐Atsu Tsi, Bamenda Hospital, Cameroon; Rogers Ajeh, Mark Benwi, Anastase Dzudie, Akindeh Mbuh, Marc Lionel Ngamani, Victorine Nkome, CRENC & Douala General Hospital, Cameroon; Djenabou Amadou, Eric Ngassam, Eric Walter Pefura Yone, Jamot Hospital, Cameroon; Alice Ndelle Ewanoge, Norbert Fuhngwa, Chris Moki, Denis Nsame Nforniwe, Limbe Regional Hospital, Cameroon; Catherine Akele, Faustin Kitetele, Patricia Lelo, Martine Tabala, Kalembelembe Pediatric Hospital, Democratic Republic of Congo; Emile Wemakoy Okitolonda, Landry Wenzi, Kinshasa School of Public Health, Democratic Republic of Congo; Merlin Diafouka, Martin Herbas Ekat, Dominique Mahambou Nsonde, CTA Brazzaville, Republic of Congo; Adolphe Mafou, CTA Pointe‐Noire, Republic of Congo; Fidele Ntarambirwa, Bethsaida Hospital, Rwanda; Yvonne Tuyishimire, Busanza Health Center, Rwanda; Theogene Hakizimana, Gahanga Health Center, Rwanda; Josephine Ayinkamiye, Gikondo Health Center, Rwanda; Sandrine Mukantwali, Kabuga Health Center, Rwanda; Henriette Kayitesi, Olive Uwamahoro, Kicukiro Health Center, Rwanda; Viateur Habumuremyi, Jules Ndumuhire, Masaka Health Center, Rwanda; Joyce Mukamana, Yvette Ndoli, Oliver Uwamahoro, Nyarugunga Health Center, Rwanda; Gallican Kubwimana, Pacifique Mugenzi, Benjamin Muhoza, Athanase Munyaneza, Emmanuel Ndahiro, Diane Nyiransabimana, Jean d'Amour Sinayobye, Vincent Sugira, Rwanda Military Hospital, Rwanda; Chantal Benekigeri, Gilbert Mbaraga, WE‐ACTx Health Center, Rwanda.

#### Coordinating and data centres

Adebola Adedimeji, Kathryn Anastos, Madeline Dilorenzo, Lynn Murchison, Jonathan Ross, Albert Einstein College of Medicine, USA; Diane Addison, Margaret Baker, Ellen Brazier, Heidi Jones, Elizabeth Kelvin, Sarah Kulkarni, Grace Liu, Denis Nash, Matthew Romo, Olga Tymejczyk, Institute for Implementation Science in Population Health, Graduate School of Public Health and Health Policy, City University of New York (CUNY), USA; Batya Elul, Columbia University, USA; Xiatao Cai, Don Hoover, Hae‐Young Kim, Chunshan Li, Qiuhu Shi, Data Solutions, USA; Robert Agler, Kathryn Lancaster, Marcel Yotebieng, Ohio State University, USA; Mark Kuniholm, University at Albany, State University of New York, USA; Andrew Edmonds, Angela Parcesepe, University of North Carolina at Chapel Hill, USA; Olivia Keiser, University of Geneva; Stephany Duda; Vanderbilt University School of Medicine, USA; April Kimmel, Virginia Commonwealth University School of Medicine, USA; Margaret McNairy, Weill Cornell Medical Center.

### East Africa IeDEA

Research reported in this publication was supported by the National Institute Of Allergy And Infectious Diseases (NIAID), Eunice Kennedy Shriver National Institute Of Child Health & Human Development (NICHD), National Institute On Drug Abuse (NIDA), National Cancer Institute (NCI), and the National Institute of Mental Health (NIMH), in accordance with the regulatory requirements of the National Institutes of Health under Award Number U01AI069911East Africa IeDEA Consortium. The content is solely the responsibility of the authors and does not necessarily represent the official views of the National Institutes of Health.

#### Site investigators and cohorts and data managers

Diero L, Ayaya S, Sang E, MOI University, AMPATH Plus, Eldoret, Kenya; Bukusi E, Charles Karue Kibaara, Elisheba Mutegi, KEMRI (Kenya Medical Research Institute), Kisumu, Kenya; John Ssali, Mathew Ssemakadde, Masaka Regional Referral Hospital, Masaka, Uganda; Mwebesa Bosco Bwana, Michael Kanyesigye, Mbarara University of Science and Technology (MUST), Mbarara, Uganda; Barbara Castelnuovo; John Michael Matovu, Infectious Diseases Institute (IDI), Mulago, Uganda; Fred Nalugoda, Francis X. Wasswa, Rakai Health Sciences Program, Kalisizo, Uganda; G.R. Somi, Joseph Nondi, NACP (National AIDS Control Program) Dar es Salaam, Tanzania; Rita Elias Lyamuya, Francis Mayanga, Morogoro Regional Hospital, Morogoro, Tanzania; Kapella Ngonyani, Jerome Lwali, Tumbi Regional Hospital, Pwani, Tanzania; Mark Urassa, Denna Michael, Richard Machemba, National Institute for Medical Research (NIMR), Kisesa HDSS, Mwanza, Tanzania; Kara Wools‐Kaloustian, Constantin Yiannoutsos, Rachel Vreeman, Beverly Musick, Indiana University School of Medicine, Indiana University, Indianapolis, IN, USA; Batya Elul, Columbia University, New York City, NY, USA; Jennifer Syvertsen, Ohio State University, Columbus, OH, USA; Rami Kantor, Brown University/Miriam Hospital, Providence, RI, USA; Jeffrey Martin, Megan Wenger, Craig Cohen, Jayne Kulzer, University of California, San Francisco, CA, USA; Paula Braitstein, University of Toronto, Toronto, Canada.

### IeDEA Southern Africa

Research reported in this publication was supported by the National Institute of Allergy and Infectious Diseases of the National Institutes of Health under Award Number U01AI069924. The content is solely the responsibility of the authors and does not necessarily represent the official views of the National Institutes of Health.

#### Site investigators and cohorts

Gary Maartens, Aid for AIDS, South Africa; Michael Vinikoor, Centre for Infectious Disease Research in Zambia (CIDRZ), Zambia; Monique van Lettow, Dignitas, Malawi; Robin Wood, Gugulethu ART Programme, South Africa; Nosisa Sipambo, Harriet Shezi Clinic, South Africa; Frank Tanser, Africa Centre for Health & Population Studies (Hlabisa), South Africa; Andrew Boulle, Khayelitsha ART Programme, South Africa; Geoffrey Fatti, Kheth'Impilo, South Africa; Sam Phiri, Lighthouse Clinic, Malawi; Cleophas Chimbetete, Newlands Clinic, Zimbabwe; Karl Technau, Rahima Moosa Mother and Child Hospital, South Africa; Brian Eley, Red Cross Children's Hospital, South Africa; Josephine Muhairwe, SolidarMed Lesotho; Anna Jores, SolidarMed Mozambique; Cordelia Kunzekwenyika, SolidarMed Zimbabwe, Matthew P Fox, Themba Lethu Clinic, South Africa; Hans Prozesky, Tygerberg Academic Hospital, South Africa.

#### Data centres

Nina Anderegg, Marie Ballif, Lina Bartels, Julia Bohlius, Frédérique Chammartin, Benedikt Christ, Cam Ha Dao Ostinelli, Matthias Egger, Lukas Fenner, Per von Groote, Andreas Haas, Taghavi Katayoun, Eliane Rohner, Lilian Smith, Adrian Spörri, Gilles Wandeler, Elizabeth Zaniewski, Kathrin Zürcher, Institute of Social and Preventive Medicine, University of Bern, Switzerland; Andrew Boulle, Morna Cornell, Mary‐Ann Davies, Victoria Iyun, Leigh Johnson, Mmamapudi Kubjane, Nicola Maxwell, Tshabakwane Nembandona, Patience Nyakato, Ernest Mokotoane, Gem Patten, Michael Schomaker, Priscilla Tsondai, Renee de Waal, School of Public Health and Family Medicine, University of Cape Town, South Africa.

### IeDEA West Africa

Research reported in this publication was supported by the US National Institutes of Health (NIAID, NICHD, NCI and NIMH) under Award Number U01AI069919 (PI: Dabis). The content is solely the responsibility of the authors and does not necessarily represent the official views of the National Institutes of Health.

#### Site investigators and cohorts

Paediatric cohorts: Sikiratou Adouni Koumakpai‐Adeothy,_CNHU, Cotonou, Benin; Lorna Awo Renner, Korle Bu Hospital, Accra, Ghana; Sylvie Marie N'Gbeche, ACONDA CePReF, Abidjan, Ivory Coast; Clarisse Amani Bosse, ACONDA_MTCT+, Abidjan, Ivory Coast; Kouadio Kouakou, CIRBA, Abidjan, Cote d'Ivoire; Madeleine Amorissani Folquet, CHU de Cocody, Abidjan, Cote d'Ivoire; François Tanoh Eboua, CHU de Yopougon, Abidjan, Cote d'Ivoire; Fatoumata Dicko Traore, Hopital Gabriel Toure, Bamako, Mali; Elom Takassi, CHU Sylvanus Olympio, Lomé, Togo.

#### Coordinating and data centres

François Dabis, Elise Arrive, Eric Balestre, Renaud Becquet, Charlotte Bernard, Shino Chassagne Arikawa, Alexandra Doring, Antoine Jaquet, Karen Malateste, Elodie Rabourdin, Thierry Tiendrebeogo, ADERA, ISPED & INSERM U1219, Bordeaux, France.

Sophie Desmonde, Julie Jesson, Valeriane Leroy, Inserm 1027, Toulouse, France Didier Koumavi Ekouevi, Jean‐Claude Azani, Patrick Coffie, Abdoulaye Cissé, Guy Gnepa, Apollinaire Horo, Christian Kouadio, Boris Tchounga, PACCI, CHU Treichville, Abidjan, Côte d'Ivoire.
