# Supplementary material for: Mortality and losses to follow‐up among adolescents living with HIV in the IeDEA global cohort collaboration
Source: J Int AIDS Soc. 2018 Dec 13;21(12):e25215. doi: 10.1002/jia2.25215 (PMC6291755; doi:10.1002/jia2.25215)
Supplement: Supplementary file 1 — Table S1. Characteristics at antiretroviral initiation for 44,922 adolescents who had a care visit at 10 to 19 years of age at an IeDEA site between 2003 and 2016 Table S2. Outcomes*, by region and age at first clinic visit (Group A, first visit <15 years of age; Group B, first visit ≥15 years of age) Table S3. Outcomes*, by region and age at first clinic visit (Group A, first visit <10 years of age; Group B, first visit ≥10 years of age) [file JIA2-21-e25215-s001.docx]

**Supplemental Table S1. Characteristics at antiretroviral initiation for 44,922 adolescents who had a care visit at 10-19 years of age at an IeDEA site between 2003-2016.**

| **Characteristics** | **All adolescents**  **N=44,922** | **First visit <age 15**  **N=35,261** | **First visit ≥age 15**  **N=9661** | ***p*-value** |
| --- | --- | --- | --- | --- |
| **Sex** |  |  |  | <0.001 |
| Male | 19,087 (42.5) | 16,917 (48.0) | 2170 (22.5) |  |
| Female | 25,688 (57.2) | 18,198 (51.6) | 7490 (77.5) |  |
| Unknown | 147 (0.3) | 146 (0.4) | 1 (0.01) |  |
| **Age at antiretroviral initiation (years)** |  |  |  | NA |
| <5 | 3871 (8.6) | 3871 (11.0) | 0 |  |
| 5-9 | 14,003 (31.2) | 13,989 (39.7) | 14 (0.1) |  |
| 10-14 | 16,397 (36.5) | 16,330 (46.3) | 67 (0.7) |  |
| ≥15 | 10,651 (23.7) | 1071 (3.0) | 9580 (99.2) |  |
| Median (IQR) | 11.1 (8.1, 14.7) | 9.9 (7.2, 12.2) | 17.5 (16.4, 18.4) |  |
| Mean (SD) | 11.3 (4.46) | 9.6 (3.46) | 17.4 (1.27) |  |
| **Severe immune deficiency^a^** |  |  |  | <0.001 |
| Yes | 16,804 (57.5) | 13,568 (59.4) | 3236 (50.5) |  |
| No | 12,431 (42.5) | 9261 (40.6) | 3170 (49.5) |  |
| **CD4 percentage^b^** |  |  |  | <0.001 |
| <10% | 7070 (15.7) | 6055 (17.2) | 1015 (10.5) |  |
| 10-14% | 4232 (9.4) | 3656 (10.4) | 576 (6.0) |  |
| 15-24% | 5022 (11.2) | 4145 (11.8) | 877 (9.1) |  |
| ≥25% | 2508 (5.6) | 1867 (5.3) | 641 (6.6) |  |
| Unknown | 26,090 (58.1) | 19,538 (55.4) | 6552 (67.8) |  |
| Median (IQR) percentage | 12 (6-19) | 12 (6-18) | 14 (7-23) | <0.001 |
| Mean (SD) | 14 (9.8) | 13 (9.5) | 16 (11.0) |  |
| **CD4 count (cells/mm^3^)^b^** |  |  |  | <0.001 |
| <200 | 11,592 (25.8) | 8788 (24.9) | 2804 (29.0) |  |
| 200-349 | 7435 (16.6) | 5406 (15.3) | 2029 (21.0) |  |
| 350-499 | 3868 (8.6) | 3100 (8.8) | 768 (8.0) |  |
| ≥500 | 5446 (12.1) | 4663 (13.2) | 783 (8.1) |  |
| Unknown | 16,581 (36.9) | 13,304 (37.7) | 3277 (33.9) |  |
| Median (IQR) | 251 (108, 423) | 260 (107, 450) | 229 (110, 346) | <0.001 |
| Mean (SD) | 319 (302.8) | 334 (321.4) | 267 (219.8) |  |
| **HIV viral load, copies/mL** |  |  |  | <0.001 |
| <50 | 321 (0.7) | 281 (0.8) | 40 (0.4) |  |
| 50-399 | 188 (0.4) | 147 (0.4) | 41 (0.4) |  |
| 400-999 | 100 (0.2) | 88 (0.3) | 12 (0.1) |  |
| 1000-9999 | 582 (1.3) | 506 (1.4) | 76 (0.8) |  |
| ≥10,000 | 3914 (8.7) | 3590 (10.2) | 324 (3.4) |  |
| Unknown | 39,817 (88.7) | 30,649 (86.9) | 9168 (94.9) |  |
| Median log10 (IQR) HIV-RNA | 4.9 (4.1, 5.4) | 4.9 (4.1, 5.4) | 4.7 (3.6, 5.2) | <0.001 |
| Mean (SD) | 4.6 (1.31) | 4.6 (1.29) | 4.3 (1.40) |  |
| **WHO/CDC clinical stage** |  |  |  | <0.001 |
| WHO stage 1/ CDC stage N | 2129 (4.7) | 1291 (3.7) | 838 (8.7) |  |
| WHO stage 2/ CDC stage A | 3086 (6.9) | 2552 (7.2) | 534 (5.5) |  |
| WHO stage 3/ CDC stage B | 3959 (8.8) | 3322 (9.4) | 637 (6.6) |  |
| WHO stage 4/ CDC stage C | 2598 (5.8) | 2351 (6.7) | 247 (2.6) |  |
| Not documented | 33,150 (73.8) | 25,745 (73.0) | 7405 (76.7) |  |
| **Weight-for-age z-score score** |  |  |  | <0.001 |
| <-3 | 8885 (19.8) | 7641 (21.7) | 1244 (12.9) |  |
| -3 ≤ to <-2 | 6566 (14.6) | 5834 (16.6) | 732 (7.6) |  |
| -2 ≤ to <-1 | 7501 (16.7) | 6396 (18.1) | 1105 (11.4) |  |
| ≥-1 | 9060 (20.2) | 5827 (16.5) | 3233 (33.5) |  |
| Unknown | 12,910 (28.7) | 9563 (27.1) | 3347 (34.6) |  |
| Median (IQR) | -1.9 (-3.2, -0.9) | -2.1 (-3.3, -1.1) | -0.9 (-2.5, 0.1) | <0.001 |
| Mean (SD) | -2.1 (1.89) | -2.3 (1.79) | -1.5 (2.11) |  |
| **Height-for-age z-score score** |  |  |  | <0.001 |
| <-3 | 6176 (13.8) | 5729 (16.3) | 447 (4.6) |  |
| -3 ≤ to <-2 | 6258 (13.9) | 5666 (16.1) | 592 (6.1) |  |
| -2 ≤ to <-1 | 6584 (14.7) | 5282 (15.0) | 1302 (13.5) |  |
| ≥-1 | 6339 (14.1) | 4092 (11.6) | 2247 (23.3) |  |
| Unknown | 19,565 (43.6) | 14,492 (41.1) | 5073 (52.5) |  |
| Median (IQR) | -2.0 (-3.0, -1.0) | -2.2 (-3.1, -1.3) | -1.0 (-1.9, -0.3) | <0.001 |
| Mean (SD) | -2.0 (1.5) | -2.2 (1.5) | -1.2 (1.4) |  |
| **First antiretroviral regimen^c^** |  |  |  | <0.001 |
| 3-ART-NNRTI | 37,435 (83.3) | 29,023 (82.3) | 8412 (87.1) |  |
| 3-ART-PI | 1659 (3.7) | 1256 (3.6) | 403 (4.2) |  |
| 3-ART-NNRTI\PI | 41 (0.1) | 34 (0.1) | 7 (0.1) |  |
| 3-ART-other | 127 (0.3) | 100 (0.3) | 27 (0.3) |  |
| Non-3-ART;  mono\dual | 5660 (12.6) | 4848 (13.7) | 812 (8.4) |  |
| **Antiretroviral initiation year** |  |  |  | <0.001 |
| 2003-2006 | 9882 (22) | 9107 (25.8) | 775 (8.0) |  |
| 2007-2010 | 17,342 (38.6) | 14,826 (42.1) | 2516 (26.0) |  |
| 2011-2016 | 17,698 (39.4) | 11,328 (32.1) | 6370 (65.9) |  |

Notes:

IQR: interquartile range, SD: standard deviation, 3-ART: antiretroviral therapy regimen of three or more drugs, NRTI: nucleoside reverse transcriptase inhibitors, NNRTI: non-nucleoside reverse transcriptase inhibitor, PI: protease inhibitor, mono/dual: one or two antiretroviral drugs. Data are presented as N (%) unless otherwise noted. We used the 1977 WHO growth curve for weight-for-age z-score (more recent weight curves are limited to children age <10 years) and the 2007 WHO growth curve for height-for-age z-score.

^a^ Severe immune deficiency was defined based on the age-specific CD4 thresholds provided in the 2006 WHO global HIV treatment guidelines.

^b^ Reported for all who had the test irrespective of age at antiretroviral initiation. Of 18,832 with CD4 percentage available, 3048 (16%) aged <5 years and of 28,341 with CD4 count, 24,818 (88%) aged ≥5 years.

**^c^** 3-ART represents triple-drug regimens. The drug class following that term denotes where one of the drugs included either an NNRTI, PI, or both classes; “other” represents triple-drug regimens without an NNRTI or PI. Non-3-ART represents regimens with fewer than three individual antiretroviral drugs.

## Supplemental Table S2. Outcomes^*^, by region and age at first clinic visit (Group A, first visit <15 years of age; Group B, first visit ≥15 years of age).

| **Region** | **Total number**  **N=61,242** | **Known deaths**  **N=2395** | **LTFU**  **N=18,613** | **Transferred**  **N=4978** |
| --- | --- | --- | --- | --- |
| Asia-Pacific | 2508 | 89 (3.6) | 68 (2.7) | 454 (18.1) |
| *Group A* | *2460* | *86 (3.5)* | *66 (2.7)* | *443 (18.0)* |
| *Group B* | *48* | *3 (6.3)* | *2 (0.03)* | *11 (22.9)* |
| CCASAnet | 1728 | 124 (7.2) | 740 (42.8) | 56 (3.2) |
| *Group A* | *1156* | *96 (8.3)* | *580 (50.2)* | *50 (4.3)* |
| *Group B* | *572* | *28 (3.9)* | *160 (28.0)* | *6 (0.7)* |
| Central Africa | 2143 | 71 (3.3) | 477 (22.3) | 98 (4.6) |
| *Group A* | *1858* | *65 (3.5)* | *387 (20.8)* | *89 (4.8)* |
| *Group B* | *285* | *6 (2.1)* | *90 (31.6)* | *9 (3.2)* |
| East Africa | 10,767 | 449 (4.2) | 2649 (24.6) | 555 (5.2) |
| *Group A* | *7085* | *298 (4.2)* | *1527 (21.6)* | *414 (5.8)* |
| *Group B* | *3682* | *151 (4.1)* | *1122 (30.5)* | *141 (3.8)* |
| South Africa | 15,494 | 236 (1.5) | 6196 (40.0) | 1246 (8.0) |
| *Group A* | *9789* | *181 (1.9)* | *3741 (38.2)* | *1155 (11.8)* |
| *Group B* | *5705* | *55 (1.0)* | *2455 (43.0)* | *91 (1.6)* |
| Southern Africa | 25,102 | 1177 (4.7) | 7943 (31.6) | 2260 (9.0) |
| *Group A* | *16,957* | *728 (4.3)* | *4722 (27.9)* | *1650 (9.7)* |
| *Group B* | *8145* | *449 (5.5)* | *3221 (39.6)* | *610 (7.5)* |
| Western Africa | 3500 | 249 (7.1) | 540 (15.4) | 309 (8.8) |
| *Group A* | *2833* | *216 (7.6)* | *369 (13.0)* | *292 (10.3)* |
| *Group B* | *667* | *33 (5.0)* | *171 (25.6)* | *17 (2.6)* |
| **TOTAL** | ***61,242*** | **2395 (3.9)** | **18,613 (30.4)** | **4978 (8.1)** |
| *Group A* | *42,138* | *1670 (4.0)* | *11,392 (27.0)* | *4093 (9.7)* |
| *Group B* | *19,104* | *725 (3.8)* | *7221 (37.8)* | *885 (4.6)* |

Notes:

LTFU: lost to follow-up. ^*^The first outcome that occurred during the first year following the last visit.

## Supplemental Table S3. Outcomes^*^, by region and age at first clinic visit (Group A, first visit <10 years of age; Group B, first visit ≥10 years of age).

| **Region** | **Total number**  **N=61,242** | **Known deaths**  **N=2395** | **LTFU**  **N=18,613** | **Transferred**  **N=4978** |
| --- | --- | --- | --- | --- |
| Asia-Pacific | 2508 | 89 (3.6) | 68 (2.7) | 454 (18.1) |
| *Group A* | *1852* | *30 (1.6)* | *41 (2.2)* | *247 (13.3)* |
| *Group B* | *656* | *59 (9.0)* | *27 (4.1)* | *207 (31.6)* |
| CCASAnet | 1728 | 124 (7.2) | 740 (42.8) | 56 (3.2) |
| *Group A* | *692* | *25 (3.6)* | *378 (54.6)* | *31 (4.5)* |
| *Group B* | *1036* | *99 (9.6)* | *362 (34.9)* | *25 (2.4)* |
| Central Africa | 2143 | 71 (3.3) | 477 (22.3) | 98 (4.6) |
| *Group A* | *1107* | *21 (1.9)* | *156 (14.1)* | *54 (4.9)* |
| *Group B* | *1036* | *50 (4.8)* | *321 (31.0)* | *44 (4.3)* |
| East Africa | 10,767 | 449 (4.2) | 2649 (24.6) | 555 (5.2) |
| *Group A* | *3812* | *86 (2.3)* | *445 (11.7)* | *194 (5.1)* |
| *Group B* | *6955* | *363 (5.2)* | *2204 (31.7)* | *361 (5.2)* |
| South Africa | 15,494 | 236 (1.5) | 6196 (40.0) | 1246 (8.0) |
| *Group A* | *5131* | *38 (0.7)* | *1525 (29.7)* | *745 (14.5)* |
| *Group B* | *10,363* | *198 (1.9)* | *4671 (45.1)* | *501 (4.8)* |
| Southern Africa | 25,102 | 1177 (4.7) | 7943 (31.6) | 2260 (9.0) |
| *Group A* | *7659* | *134 (1.8)* | *1188 (15.5)* | *624 (8.2)* |
| *Group B* | *17,443* | *1043 (6.0)* | *6755 (38.7)* | *1636 (9.4)* |
| Western Africa | 3500 | 249 (7.1) | 540 (15.4) | 309 (8.8) |
| *Group A* | *1915* | *89 (4.7)* | *190 (9.9)* | *141 (7.4)* |
| *Group B* | *1585* | *160 (10.1)* | *350 (22.1)* | *168 (10.6)* |
| **TOTAL** | ***61,242*** | **2395 (3.9)** | **18,613 (30.4)** | **4978 (8.1)** |
| *Group A* | *22,168* | *423 (1.9)* | *3923 (17.7)* | *2036 (9.2)* |
| *Group B* | *39,074* | *1972 (5.1)* | *14,690 (37.6)* | *2942 (7.5)* |

Notes:

LTFU: lost to follow-up. ^*^The first outcome that occurred during the first year following the last visit.
